# Use of Hybrid PEDOT:PSS/Metal Sulfide Quantum Dots for a Hole Injection Layer in Highly Efficient Green Phosphorescent Organic Light-Emitting Diodes

**DOI:** 10.3389/fchem.2021.657557

**Published:** 2021-04-29

**Authors:** Wenqing Zhu, Kuangyu Ding, Chen Yi, Ruilin Chen, Bin Wei, Lu Huang, Jun Li

**Affiliations:** ^1^ School of Materials Science and Engineering, Shanghai University, Shanghai, China; ^2^ School of Mechatronic Engineering and Automation, Key Laboratory of Advanced Display and System Applications, Ministry of Education, Shanghai University, Shanghai, China

**Keywords:** phosphorescent organic light-emitting diodes, metal sulfide QDs, hybrid hole injection layer, PEDOT:PSS, ZnS, MoS_2_

## Abstract

In this study, we have synthesized the molybdenum sulfide quantum dots (MoS_2_ QDs) and zinc sulfide quantum dots (ZnS QDs) and demonstrated a highly efficient green phosphorescent organic light-emitting diode (OLED) with hybrid poly (3,4-ethylenedioxythiophene)/poly (styrenesulfonate) (PEDOT:PSS)/QDs hole injection layer (HIL). The electroluminescent properties of PEDOT:PSS and hybrid HIL based devices were explored. An optimized OLED based on the PEDOT:PSS/MoS_2_ QDs HIL exhibited maximum current efficiency (CE) of 72.7 cd A^−1^, which shows a 28.2% enhancement as compared to counterpart with single PEDOT:PSS HIL. The higher device performance of OLED with hybrid HIL can be attributed to the enhanced hole injection capacity and balanced charge carrier transportation in the OLED devices. The above analysis illustrates an alternative way to fabricate the high efficiency OLEDs with sulfide quantum dots as a HIL.

## Introduction

New-generation organic light-emitting diodes (OLEDs) have attracted huge research interests due to their unique properties such as high color purity, light weight and flexibility (Liao et al., 2004; Wen et al., 2005; Wang et al., 2018; Wang et al., 2020). One of the most important issue for the OLEDs application in industry is the device efficiency. The effective charge injection and transportation, and exciton confinement in the emitting layer are the key parameters to achieve highly efficient OLEDs (Lee et al., 1999; Wang et al., 2008; Song et al., 2018; Zhao et al., 2020). To date, many research groups have used the solution processed hole injection layer (HIL) to improve the device performance and to decrease the fabrication costs (Li et al., 2018; Hu et al., 2020; Wang et al., 2020). To obtain the highly efficient OLEDs, the overall requirements on solution processed HIL should possess excellent optical and electrical characteristics such as high transparency and conductivity, as well as the low surface roughness (Zhao et al., 2016; McEwan et al., 2018; Feng et al., 2020). Therefore, the synthesis and development of solution processed hole injection materials are very important to achieve high-performance devices.

The traditional poly (3,4-ethylenedioxythiophene)/poly (styrenesulfonate) (PEDOT:PSS) is widely used as a solution processed HIL in organic electronics (Benor et al., 2010; Salsberg and Aziz, 2019). However, its acidic property and easily absorption for water in the air significantly deteriorate the device performance. The traditional molybdenum oxide (MoO_x_) is also employed in the OLED devices as an effective HIL due to its matched work function with ITO electrode and low surface roughness and high transparency. However, the MoO_x_ as HIL is normally prepared by using vacuum thermal evaporation method which is not a good candidate for low-cost OLED preparation. More importantly, to date, there is seldom reports related to the solution-processed MoO_x_ in electronic devices because of the limited solubility. Recently, solution processed sulfide quantum dots (QDs) such as zinc sulfide QDs (Zn QDs), molybdenum sulfide QDs (MoS_2_ QDs), and others as HILs/HTLs have been reported as an effective way to increase charge injection due to their unique properties, such as high solubility, tunable work function and low cost, resulting in the enhanced devices performance (Kim et al., 2015; Lenkeviciute et al., 2015; Xie et al., 2019). In our previous work, we have reported a highly efficient inverted OLEDs by using the solution processed ZnS QDs as an electron injection layer due to the excellent optical and electrical properties (Shi et al., 2019).

In this paper, the application of PEDOT:PSS doped with various QDs as the hybrid HIL for high-efficiency green phosphorescent OLED devices have been studied. And the hybrid PEDOT:PSS/QDs HILs were investigated based on their optical and morphological characteristics, as well as the surface energy. We found that the hybrid HIL based device revealed relatively higher device performance with the best current efficiency (CE) of 72.7 cd A^−1^, which was a 28.2% enhancement as compared to the neat PEDOT:PSS based devices.

## Experimental

### General Information

The organic functional molecules were obtained from e-Ray Optoelectronics Corp (China). The hole injection material poly (3,4-ethylenedioxythiophene)/poly (styrenesulfonate) (PEDOT:PSS) was purchased from Heraeus, Germany. Indium tin oxide (ITO, 15 Ohm per sheet, 150 nm)-coated glass substrates were ordered from CSG Holding Co. Ltd. (China). All chemicals and reagents in this work were used as received from commercial sources without further purification unless otherwise stated.

### Synthesis of Zinc Sulfide Quantum Dots and Molybdenum Sulfide Quantum Dots

**FIGURE 1:**
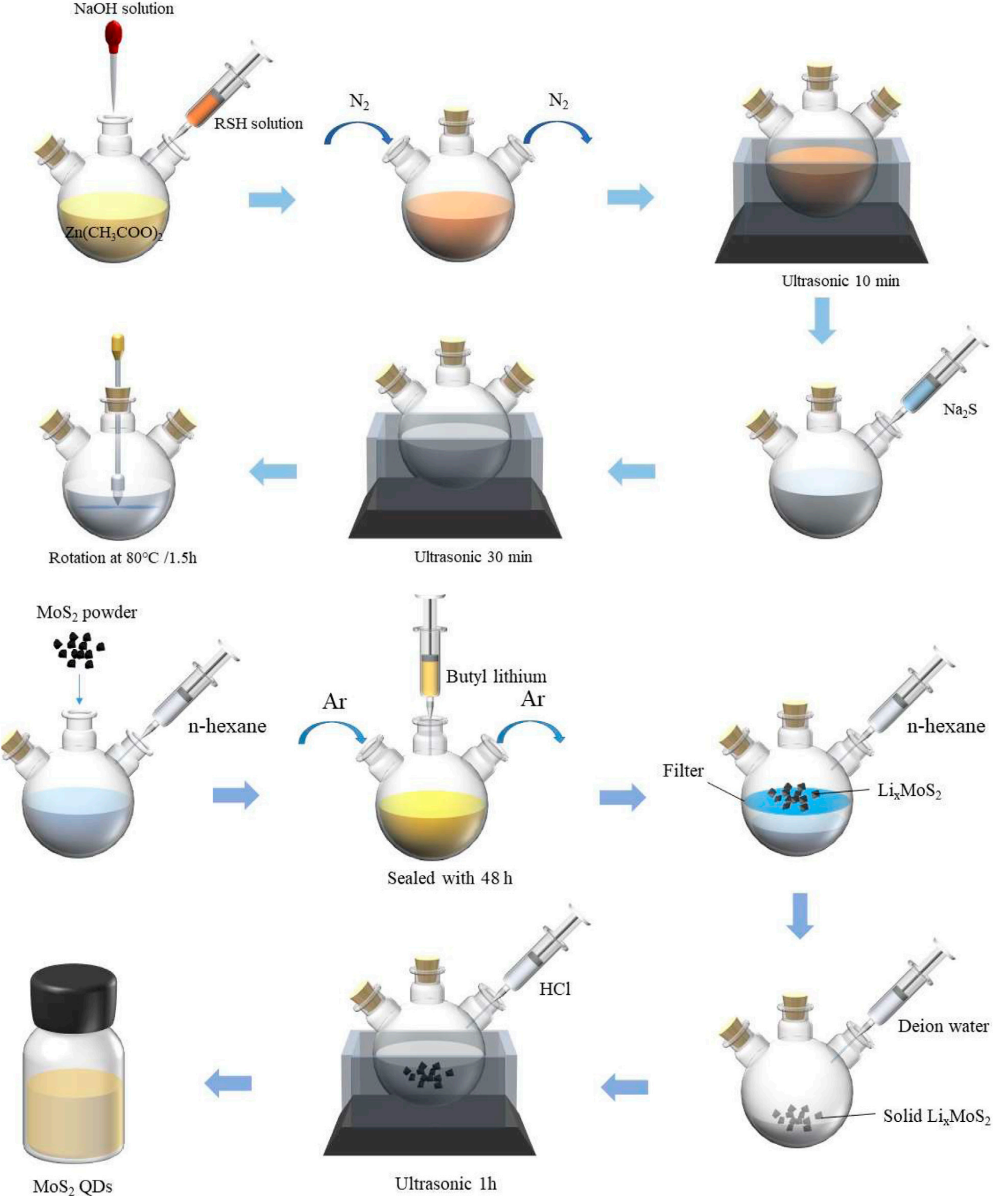
Schematic of ZnS QDs **(upper)** and MoS_2_ QD **(below)** synthesis procedure.

ZnS QDs were synthesized using our previously reported method (Shi et al., 2019). As shown in Figure 1, in brief, take out 2.4018 g Na_2_S·9H_2_O and put it into a 100 ml volumetric flask. add distilled water to 100 ml scale, mix well and keep well. Then 0.02 mol L^−1^ RSH aqueous solution and 0.1 mol L^−1^ Zn (CH_3_COO)_2_ solution was prepared. The above Na_2_S aqueous solution is the same. Add 25 ml of 0.1 mol^–1^ Zn (CH_3_COO)_2_ solution above into a three necked round bottom flask, continue to add 50 ml of 0.02 mol^−1^ ethanoic acid, and then dissolve it with 0.5 mol L^−1^ NaOH. Finally, after ultrasonic treatment for 10 min, the prepared Na_2_S aqueous solution was added rapidly to 5 ml, and then the device was ultrasonic treated for 30 min. Finally, ZnS nano quantum dots were prepared by stirring at 80°C for 1 h and 30 min.

For the synthesis of MoS_2_ QDs, firstly, taking 1 g of MoS_2_ powder into a three-necked round bottom flask, add 12 ml of n-hexane, and passed the protective gas argon into the flask. The three-necked round bottom flask was immediately sealed and allowed to stand for 48 h. The LixMoS_2_ in the intercalation was vacuum filtered. The product obtained by suction filtration was repeatedly washed with n-hexane solution to remove excess butyl lithium and organic residues. Then quickly took out LixMoS_2_ on the suction filter membrane and made it react with deionized water. The solution formed by the reaction of LixMoS_2_ with water was placed in an ultrasonic microwave instrument for auxiliary ultrasound for 1 h. The MoS_2_ flakes in the suspension would quickly produce black flocculent precipitates, and then centrifuged with deionized water for several times to adjust the pH of the solution to medium which could remove Li, Cl ions and organic residues and obtain the MoS_2_ QD solution.

### Device Fabrication

**FIGURE 2:**
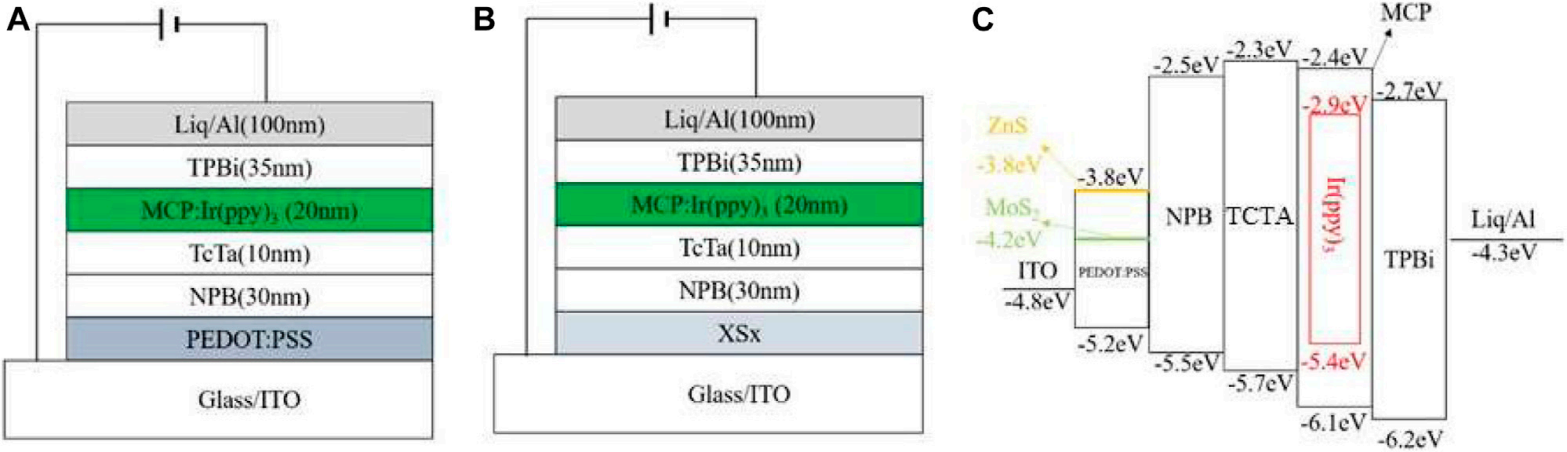
Structure of the green phosphorescent OLEDs with **(A)** neat PEDOT:PSS HIL and **(B)** hybrid HIL. **(C)** The corresponding energy level diagram of the devices.

Devices were fabricated with a configuration of ITO/PEDOT: PSS (40 nm)/NPB (30 nm)/TCTA (10 nm)/mCP: 5%Ir (ppy)_3_ (20 nm)/TPBi (35 nm)/Liq (1 nm)/Al (100 nm), as shown in Figure 2, where ITO is the anode; PEDOT: PSS is the hole injection layer; N,N′-bis(naphthalen-1-yl)-N,N′-bis(phenyl)-benzidine (NPB) and 4,4′,4″-tris(carbazol-9-yl)triphenylamine (TCTA) are the hole transporting layer. 1,3-bis(N-carbazolyl)benzene (mCP) is the host for green phosphorescent dopant; tris(2-phenyl-3-methyl-pyridine)iridium (Ir (ppy)_3_) is the green dopant; 1,3,5-tris(1-phenyl-1H-benzimidazol-2-yl)benzene (TPBi) functions as the electron transporting layer and interlayer; Liq and Al are the electron injection layer and the cathode, respectively. The patterned ITO glass substrates were first cleaned sequentially by using detergent, deionized water, acetone, isopropanol and treated with a UV-ozone environment for about 20 min. Then a 40 nm QDs doped PEDOT:PSS was spin-coated onto the ITO surface under the conditions of rotation speed of 3,000 rpm and spin coating time of 60 s. After the spin coating process was completed, the ITO covering part of the electrode was wiped off with deionized water and being baked at 130°С for 20 min under air conditions. Then the substrates were transferred into a vacuum chamber. Then, organic layers and a metal cathode layer were successively deposited by using shadow masks to finish the device fabrication in a vacuum chamber under a base pressure less than 4 × 10^−6^ mbar. The deposition rates for the organic layers and Al cathode were typically 2.0 Å s^−1^ and 5.0 Å s^−1^, respectively. The active area of OLEDs is 2 × 2 mm^2^.

### Film and Device Characterization

The transmittance spectra were recorded on a UV-2501PC spectrophotometer at room temperature. Drop shape analysis (Kino optical CA and interface tensiometer) was used to measure the contact angles of deionized (DI) water. The surface morphological images of the various HILs were analyzed in air by using AFM (Bruker, Santa Barbara, CA, USA) in a tapping mode. The EL characteristics were measured using a Keithley 2,400 source meter and a PR650 Spectra Colorimeter. The luminance and spectra of each device were measured in the direction perpendicular to the substrate. All the device characterization steps were carried out at room temperature under ambient laboratory conditions without encapsulation.

## Results and Discussion

**FIGURE 3:**
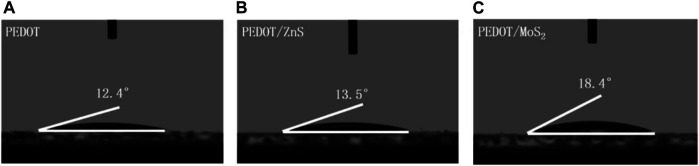
Water contact angle measurements of different HIL of **(A)** PEDOT:PSS, **(B)** PEDOT:PSS/10% ZnS QDs and **(C)** PEDOT:PSS/10% MoS_2_ QDs.

Firstly, the water contact angles of neat PEDOT:PSS and hybrid PEDOT:PSS/QDs with the concentration of 10% (v/v) on the glass substrate were measured to identify the hydrophilic and spread-ability of the resulting HILs, which could affect the film-forming property of hole transporting layer (HTL) (Yu et al., 2014; Singh et al., 2014; Huang et al., 2009; Cho et al., 2014). Figure 3 demonstrate the contact angles of water with PEDOT:PSS and PEDOT:PSS/QDs with different concentration are 12.4°, 13.5° and 18.4°, respectively. The small contact angles reveal that the hybrid PEDOT:PSS/QDs HIL has strong hydrophilic and spread-ability, resulting in a better interface and adhesion between anode and hole transporting layer (Phatak et al., 2012; Tsai et al., 2020).

**FIGURE 4:**
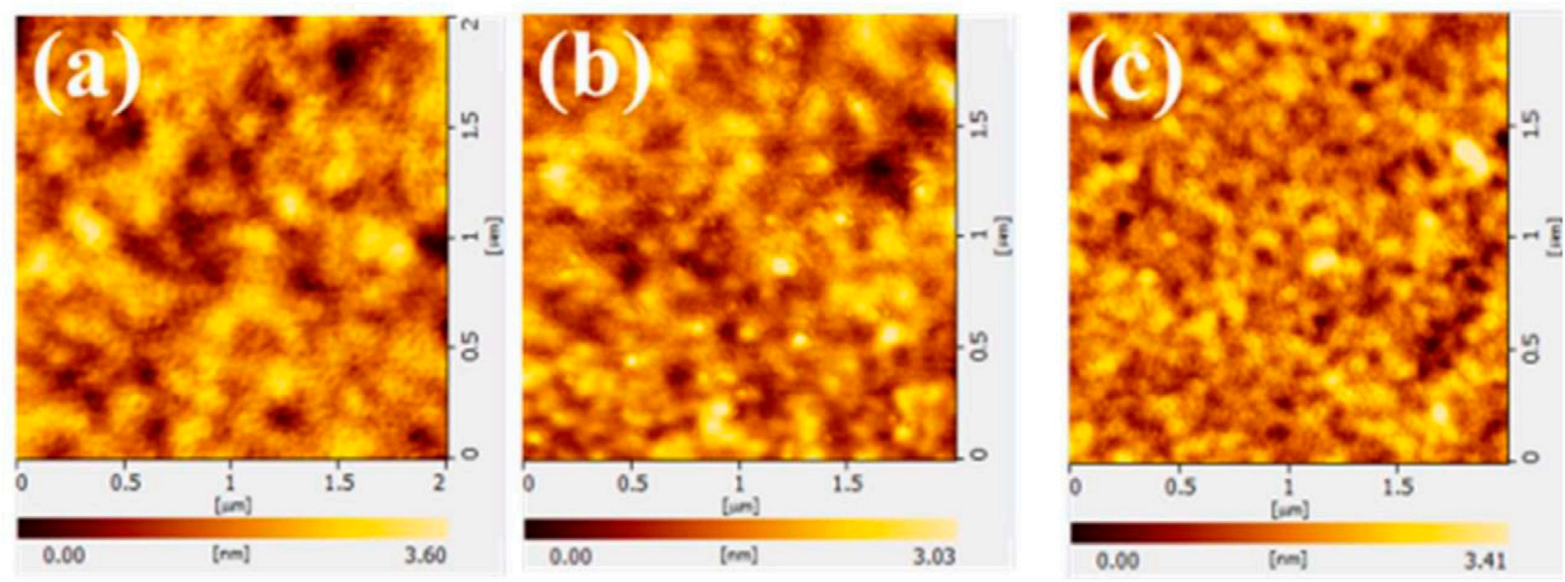
AFM test of HILs of **(A)** PEDOT:PSS, **(B)** PEDOT:PSS/10%ZnS QDs and **(C)** PEDOT:PSS/10% MoS_2_ QDs.

The OLEDs are multi thin-film structures with the overall device thickness of around 200 nm. Therefore, it is an essential requirement for OLED devices with a smooth surface on the substrate to avoid the trap states such as defects, shorts, and pinholes (Lee et al., 2018; Michels et al., 2021; Lim et al., 2008). Figure 4 shows the surface morphology profiles of glass/HIL (40 nm) observed by AFM measurements. The values of surface roughness (RMS) of neat PEDOT:PSS, PEDOT:PSS/10%ZnS QDs and PEDOT:PSS/10%MoS_2_ QDs film are 0.57, 0.47, and 0.69 nm, respectively. The hybrid HIL films exhibit a low surface roughness, which result in more efficient hole injection and consequently affect the HTL and device performance (Kim et al., 2019; Ma et al., 2019).

**FIGURE 5:**
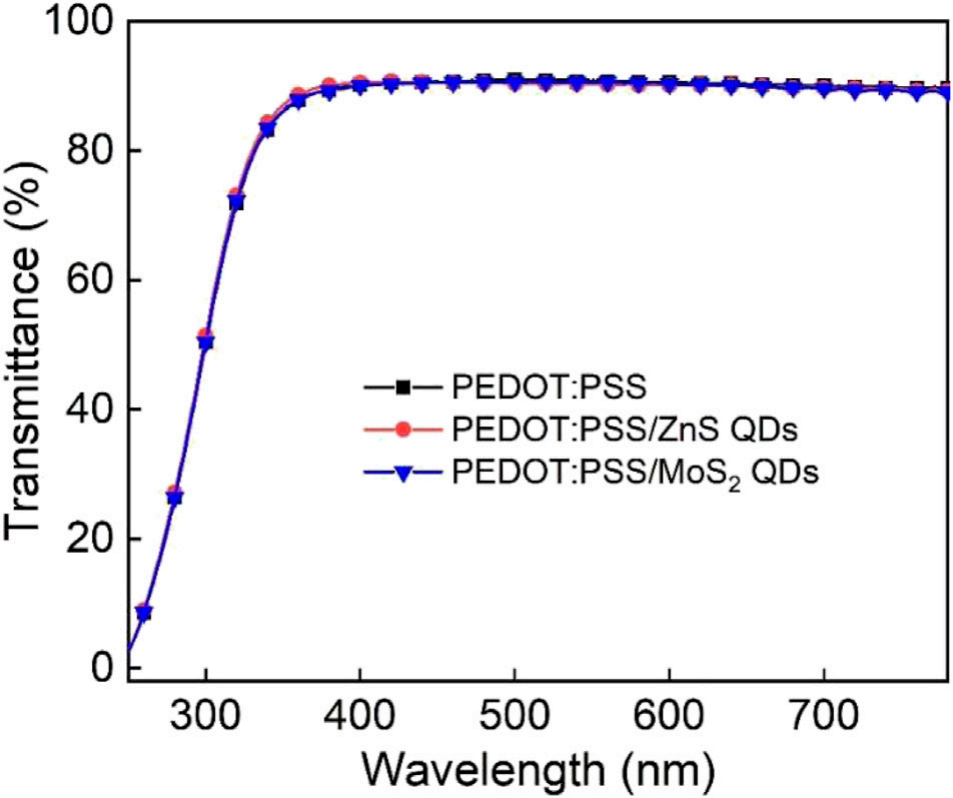
The optical transmittance spectra for neat PEDOT:PSS, PEDOT:PSS/10% ZnS QDs and PEDOT:PSS/10% MoS_2_ QDs films.

Figure 5 shows the ultraviolet visible (UV-Vis) spectra measurement with the wavelength range of 220–800 nm. The transmittances of the resulting film are over 90% in the visible region. Within the green emission spectral range, the glass substrate with hybrid PEDOT:PSS/ZnS QDs and PEDOT:PSS/MoS_2_ QDs film with the concentration of 10% yielded a higher transmittance of 93% at 525 nm which is beneficial for enhancing the light extraction in OLED devices with normal configuration (Yang et al., 2014; Chiu and Chuang, 2015). The above results demonstrate the good visible light transmittance property of sulfide QDs-based thin films.

**FIGURE 6:**
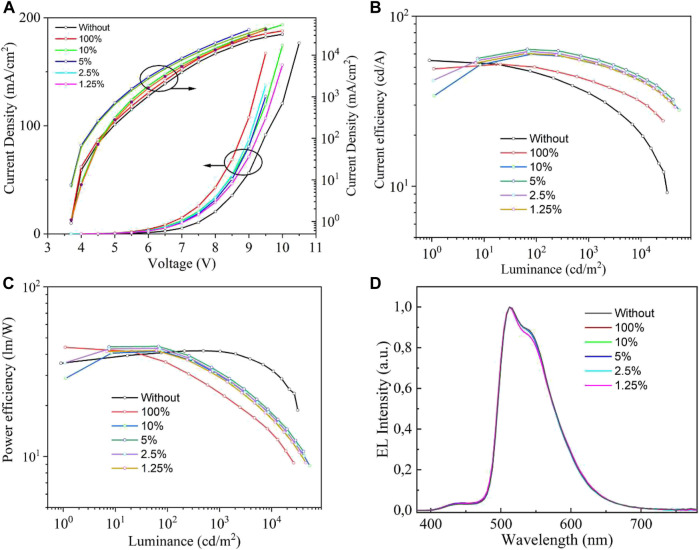
The green phosphorescent OLED device with hybrid PEDOT:PSS/ZnS QDs film as the HIL. **(A)** Current density-voltage-brightness (*J*-*V*-*L*), **(B)** Current efficiency-luminance (CE-*L*), **(C)** Power efficiency-current density (PE-*J*) and (d) EL spectra at 6 V of the resulting devices with different concentrations.

To evaluate the solution-processed hybrid PEDOT:PSS/ZnS QDs and PEDOT:PSS/MoS_2_ QDs films as the HIL, the OLED devices with normal configuration were fabricated using the following structure: ITO/HIL (40 nm)/NPB (30 nm)/TCTA (10 nm)/mCP: 5%Ir (ppy)_3_ (20 nm)/TPBi (35 nm)/Liq (1 nm)/Al (100 nm). Here, the device with neat PEDOT:PSS as HIL was also fabricated as the reference device. The corresponding energy level diagram of OLED devices are also depicted in Figure 2. The current density(*J*)-voltage(*V*)-brightness(*L*) properties of the OLEDs are exhibited in Figure 6 and the main electroluminescent properties are summarized in Table 1.

**TABLE 1:** Performance parameters of the green phosphorescent OLED device with the various HILs.

HIL	*V*_on_ [V]^a^	*n*_max_ (cd A^−1^)^b^	*L*_max_ (cd m^−2^)^c^	*n*_max_ (lm W^−1^)^d^
Neat PEDOT:PSS	4	55.1	35,962	41.9
PEDOT:PSS/100% ZnS	4.2	52.2	46,354	43.9
PEDOT:PSS/10% ZnS	4.3	59.7	49,005	41.6
PEDOT:PSS/5% ZnS	4.3	63.8	41,184	44.5
PEDOT:PSS/2.5% ZnS	4.3	62.1	41,844	43.3
PEDOT:PSS/1.25% ZnS	4.3	60.2	45,705	42.0
PEDOT:PSS/100% MoS_2_	3.6	55.8	37,950	40.7
PEDOT:PSS/15% MoS_2_	3.8	63.2	45,276	40.2
PEDOT:PSS/10% MoS_2_	3.6	72.7	46,354	41.5
PEDOT:PSS/5% MoS_2_	3.6	65.3	39,424	45.6
PEDOT:PSS/2.5% MoS_2_	3.8	61.1	45,320	38.3
PEDOT:PSS/1.25% MoS_2_	3.8	56.7	43,802	39.6

a The operating voltage at a brightness of 1 cd m^−2^.

b The maximum CE.

c The maximum luminance.

d The maximum PE.

The utilization of PEDOT:PSS/ZnS QDs based HILs significantly improved the device performances. From the *J*-*V* characteristics of the devices with various concentrations of QDs based HILs, the current density increased compared with the neat PEDOT:PSS based device. In addition, the device with hybrid PEDOT:PSS/ZnS QDs HILs showed a higher luminance at same current density, as shown in Figure 6A. It is also noteworthy that the maximum current efficiency of the device with hybrid PEDOT:PSS/ZnS QDs is higher than that of the device with neat PEDOT:PSS. The PEDOT:PSS/5%ZnS QDs based device showed excellent current efficiency (CE) of 63.8 cd A^−1^, which is superior to that of device with neat PEDOT:PSS of 55.1 cd A^−1^. It suggests that the hybrid PEDOT:PSS/ZnS QDs as HIL can significantly enhance hole injection capacity in comparison with the neat PEDOT:PSS HIL. The hybrid PEDOT:PSS/10%ZnS QDs based OLED reaches a maximum luminance of 49,005 cd m^−2^ at 9.5 V, among the other devices. Typical hybrid PEDOT:PSS/ZnS QDs based OLED gives emission with an EL peak of 516 nm, as shown in Figure 6D.

**FIGURE 7:**
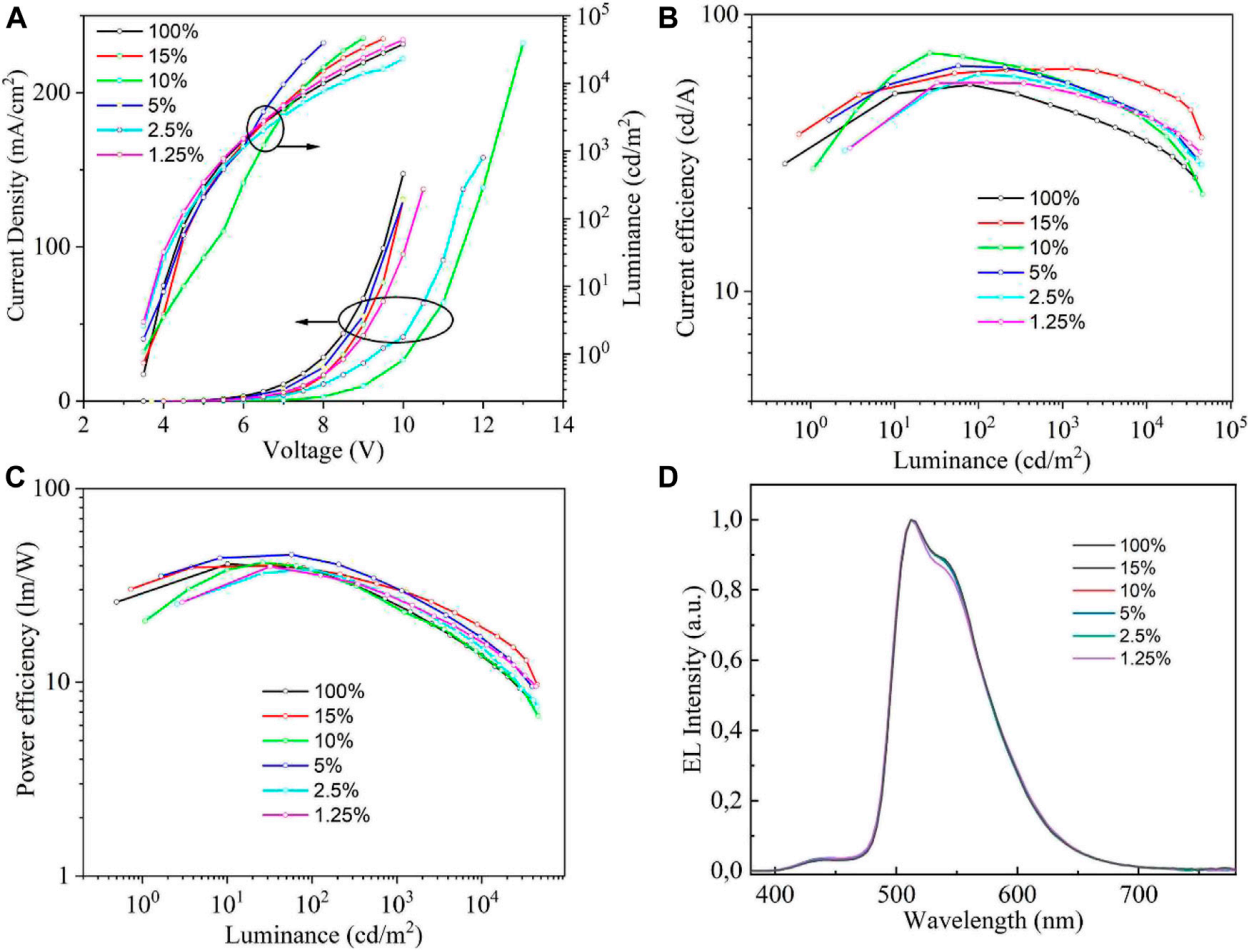
Device performance with hybrid PEDOT:PSS/MoS_2_ QDs film as the HIL. **(A)** *J-V-L*, **(B)** CE-*L*, **(C)** PE-*J* and **(D)** EL spectra at 6 V of the resulting devices with different concentrations.

Finally, we investigated the device performances based on the hybrid PEDOT:PSS/MoS_2_ QDs as the HIL. The results are shown in Figure 7, and the performance parameters are also listed in Table 1. It should be noted that device with hybrid PEDOT:PSS/10%MoS_2_ QDs shows the better performance compared with other devices. The device with the concentration of 10%MoS_2_ QDs in PEDOT:PSS exhibited maximum CE of 72.7 cd A^−1^ and maximum luminescence of 46,354 cd m^−2^ with a low turn-on voltage of 3.6 V. We also demonstrate that PEDOT:PSS doped with 10%MoS_2_ QDs forms more higher quality film with lower surface roughness, which are beneficial to form a better interface and adhesion between anode and hole transporting layer. (Phatak et al., 2012; Tsai et al., 2020).

**FIGURE 8:**
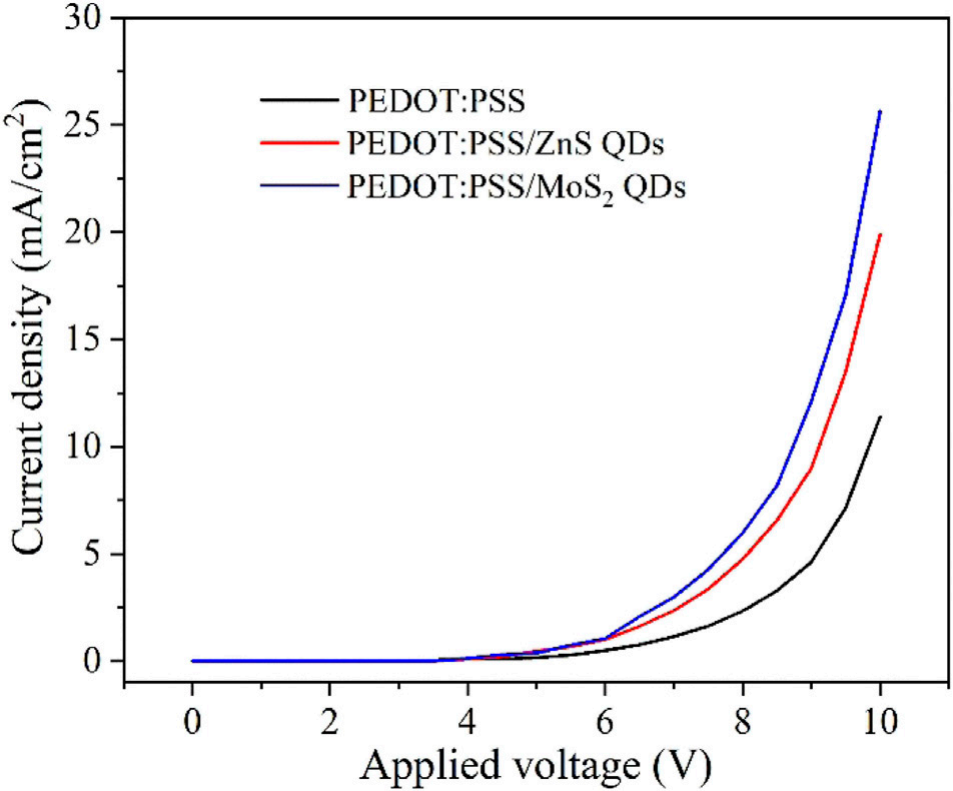
The *J-V* characteristics of hole-only devices of ITO/HIL (40 nm)/NPB (100 nm)/Al (100 nm).

To further investigate the mechanisms of the different HILs in the OLED devices, we prepared hole-only devices (HODs) with device structures of ITO/HIL (40 nm)/NPB (100 nm)/Al (100 nm) with various hole injection layers (traditional PEDOT:PSS, PEDOT:PSS/10%ZnS QD, and PEDOT:PSS/10%MoS_2_ QD) and the current density-voltage (*J*-*V*) characteristics of HODs based on the mixed HILs with the concentration of 10% were compared. The related results are shown in Figure 8. The HOD with the mixed PEDOT:PSS/QD HILs showed much higher current density than the devices with the traditional PEDOT:PSS HIL at the same driving voltages, indicating the more efficient hole injection.

The hole mobility based HOD was calculated from space charge limited current method by using the following Mott-Gurney Law equation (Tang et al., 2019):

$$J=\frac{9}{8}\varepsilon \varepsilon_{0}\mu \frac{V^{2}}{L^{3}}\exp\left(\beta \sqrt{\frac{V}{L}}\right),$$

where *ε*_0_ is the vacuum permittivity, *ε* is the relative dielectric constant, *β* is the Poole-Frankel factor, and *L* is the thickness of HIL. The mobility of NPB (1.6 × 10^−5^ cm^2^/V·S^−1^) is much lower than that of PEDOT:PSS/QD. (Blom et al., 2005; Zhao et al., 2019) Thus, in the initial SCLC region, the hole mobility of PEDOT:PSS/QD based HODs is limited by the mobility of NPB. The hole mobility of PEDOT:PSS/QD based HOD was evaluated from the linear fitting the SCLC region (based on *ε* = 11.9, *ε*_0_ = 8.85 × 10^−12^ Fm^−1^, and L = 40 nm), which results in *µ*_*1*_ = 1.748 × 10^−4^ cm^2^/V·S^−1^ (PEDOT:PSS), *µ*_*2*_ = 7.67 × 10^−4^ cm^2^/V·S^−1^ (PEDOT:PSS/ZnS QD), *µ*_*3*_ = 1.464 × 10^−3^ cm^2^/V·S^−1^ (PEDOT:PSS/MoS_2_ QD), indicating that the mixing of QD and PEDOT:PSS can improve the hole mobility compared to PEDOT:PSS based OLED obviously. This result is coincidence with device performance, which is responsible for the improvement of hole injection in OLEDs. Therefore, based on the corresponding energy level diagram of devices and the analytic results of SCLC method of HODs, the enhanced device efficiency with hybrid HILs can be attributed to the reduced hole injection barrier and charge recombination, resulting in the overall performance improvement of the resulting OLEDs. (Li and Marks, 2008; Han et al., 2015; Yadav et al., 2020)

## Conclusion

In this work, we have systematically investigated the optical and electrical properties of the ZnS and MoS_2_ QDs. We have demonstrated that hybrid PEDOT:PSS/QDs film as the HIL in conventional OLED could highly enhance hole injection from anode into organic hole transporting layer, and consequently improves the device efficiency. Furthermore, the device with hybrid HILs shows lower turn-on voltage and higher luminance because of its enhanced hole injection property. Among all the devices with various HILs, the device with hybrid PEDOT:PSS/MoS_2_ QDs HIL showed a lowest turn-on voltage of 3.6 V and the highest maximum CE of 72.7 cd A^−1^, achieving an enhancement of 28.2% than those with neat PEDOT:PSS based devices. We conclude that the improved film morphology of the hybrid HILs, balanced charge carrier injection and recombination are important factors to contribute the high performance of OLEDs.

## Data Availability

The raw data supporting the conclusions of this article will be made available by the authors, without undue reservation.

## References

[B1] Benor A. Takizawa S.-y. Pérez-Bolívar C. Anzenbacher P. Jr (2010). Efficiency Improvement of Fluorescent OLEDs by Tuning the Working Function of PEDOT:PSS Using UV-Ozone Exposure. Org. Electronics 11 (5), 938–945. 10.1016/j.orgel.2010.02.014

[B2] Blom P. W. M. Tanase C. De Leeuw D. M. Coehoorn R. (2005). Thickness Scaling of the Space-Charge-Limited Current in Poly (P-phenylene Vinylene). Appl. Phys. Lett. 86 (9), 092105. 10.1063/1.1868865

[B3] Chiu T. L. Chuang Y. T. (2015). Spectral Observations of Hole Injection with Transition Metal Oxides for an Efficient Organic Light-Emitting Diode. J. Phys. D: Appl. Phys. 48 (7), 075101. 10.1088/0022-3727/48/7/075101

[B4] Cho A. R. Kim S. H. Lee E.-W. Gwak G. Jang J. Park L. S. (2014). Flexible OLED Fabricated on Glass Fabric Reinforced Film and Performance. Mol. Crystals Liquid Crystals 602 (1), 26–33. 10.1080/15421406.2014.944365

[B5] Feng C. Zheng X. Xu R. Zhou Y. Hu H. Guo T. Li F. (2020). Highly Efficient Inkjet Printed Flexible Organic Light-Emitting Diodes with Hybrid Hole Injection Layer. Org. Electronics 85, 105822. 10.1016/j.orgel.2020.105822

[B6] Han T.-H. Song W. Lee T.-W. (2015). Elucidating the Crucial Role of Hole Injection Layer in Degradation of Organic Light-Emitting Diodes. ACS Appl. Mater. Inter. 7 (5), 3117–3125. 10.1021/am5072628 25562405

[B7] Hu Y. Song L. Zhang S. Lv Y. Lin J. Guo X. (2020). Improving the Efficiency of Multilayer Organic Light‐Emitting Transistors by Exploring the Hole Blocking Effect. Adv. Mater. Inter. 7 (17), 2000657. 10.1002/admi.202000657

[B8] Huang Z. H. Zeng X. T. Sun X. Y. Kang E. T. Fuh J. Y. H. Lu L. (2009). Influence of Electrochemical Treatment of ITO Surface on Nucleation and Growth of OLED Hole Transport Layer. Thin solid films 517 (17), 4810–4813. 10.1016/j.tsf.2009.03.020

[B10] Kim G.-E. Shin D.-K. Lee J.-Y. Park J. (2019). Effect of Surface Morphology of Slot-Die Heads on Roll-To-Roll Coatings of Fine PEDOT:PSS Stripes. Org. Electronics 66, 116–125. 10.1016/j.orgel.2018.12.033

[B9] Kim H. J. Shin M. H. Hong H. G. Song B. S. Kim S. K. Koo W. H. Kim Y. J. (2015). Enhancement of Optical Efficiency in White OLED Display Using the Patterned Photoresist Film Dispersed with Quantum Dot Nanocrystals. J. Display Technology 12 (6), 526–531.

[B12] Lee J. Kim G. Shin D.-K. Seo Y. Kim K. Park J. (2018). Improved Surface Morphology of Crosslinked Hole Transport Films by a Mixture of Polymer for OLEDs. IEEE Trans. Electron. Devices 65 (8), 3311–3317. 10.1109/ted.2018.2842130

[B11] Lee S. T. Wang Y. M. Hou X. Y. Tang C. W. (1999). Interfacial Electronic Structures in an Organic Light-Emitting Diode. Appl. Phys. Lett. 74 (5), 670–672. 10.1063/1.122982

[B13] Lenkeviciute B. Vitkus M. Juska G. Genevicius K. (2015). Hybrid OLEDs with CdSSe1-/ZnS Core-Shell Quantum Dots: An Investigation of Electroluminescence Properties. Synth. Met. 209, 343–347. 10.1016/j.synthmet.2015.08.003

[B15] Li B. Gan L. Cai X. Li X.-L. Wang Z. Gao K. (2018). An Effective Strategy toward High-Efficiency Fluorescent OLEDs by Radiative Coupling of Spatially Separated Electron-Hole Pairs. Adv. Mater. Inter. 5 (10), 1800025. 10.1002/admi.201800025

[B14] Li J. Marks T. J. (2008). Air-stable, Cross-Linkable, Hole-Injecting/transporting Interlayers for Improved Charge Injection in Organic Light-Emitting Diodes. Chem. Mater. 20 (15), 4873–4882. 10.1021/cm703689j

[B16] Liao L. S. Klubek K. P. Tang C. W. (2004). High-efficiency Tandem Organic Light-Emitting Diodes. Appl. Phys. Lett. 84 (2), 167–169. 10.1063/1.1638624

[B17] Lim S. H. Ryu G. Y. Seo J. H. Park J. H. Youn S. W. Kim Y. K. (2008). Dependence of Surface Morphology on Molecular Structure and its Influence on the Properties of OLEDs. Ultramicroscopy 108 (10), 1251–1255. 10.1016/j.ultramic.2008.04.093 18571863

[B18] Ma Y.-Y. Hua X.-C. Zhai T.-S. Li Y.-H. Lu X. Duhm S. (2019). Doped Copper Phthalocyanine via an Aqueous Solution Process for High-Performance Organic Light-Emitting Diodes. Org. Electronics 68, 236–241. 10.1016/j.orgel.2019.02.019

[B19] McEwan J. A. Clulow A. J. Nelson A. Wang R. Burn P. L. Gentle I. R. (2018). Influence of Dopant Concentration and Steric Bulk on Interlayer Diffusion in OLEDs. Adv. Mater. Inter. 5 (1), 1700872. 10.1002/admi.201700872

[B20] Michels J. J. Zhang K. Wucher P. Beaujuge P. M. Pisula W. Marszalek T. (2021). Predictive Modelling of Structure Formation in Semiconductor Films Produced by Meniscus-Guided Coating. Nat. Mater. 20 (1), 68–75. 10.1038/s41563-020-0760-2 32778811

[B21] Phatak R. Tsui T. Y. Aziz H. (2012). Dependence of Dark Spot Growth on Cathode/organic Interfacial Adhesion in Organic Light Emitting Devices. J. Appl. Phys. 111 (5), 054512. 10.1063/1.3692390

[B22] Salsberg E. Aziz H. (2019). Degradation of PEDOT:PSS Hole Injection Layers by Electrons in Organic Light Emitting Devices. Org. Electronics 69, 313–319. 10.1016/j.orgel.2019.03.009

[B23] Shi G. Zhang X. Wan M. Wang S. Lian H. Xu R. (2019). High-performance Inverted Organic Light-Emitting Diodes with Extremely Low Efficiency Roll-Off Using Solution-Processed ZnS Quantum Dots as the Electron Injection Layer. RSC Adv. 9 (11), 6042–6047. 10.1039/c8ra10290b PMC9060893 35517305

[B24] Singh A. Nehm F. Müller-Meskamp L. Hoßbach C. Albert M. Schroeder U. (2014). OLED Compatible Water-Based Nanolaminate Encapsulation Systems Using Ozone Based Starting Layer. Org. Electronics 15 (10), 2587–2592. 10.1016/j.orgel.2014.07.024

[B25] Song C. Hu Z. Luo Y. Cun Y. Wang L. Ying L. (2018). Organic/Inorganic Hybrid EIL for All-Solution-Processed OLEDs. Adv. Electron. Mater. 4 (2), 1700380. 10.1002/aelm.201700380

[B26] Tang X. Xiao S. Fu Q. Chen Y. Hu T. (2019). Incorporation of Two Electron Acceptors to Improve the Electron Mobility and Stability of Perovskite Solar Cells. J. Mater. Chem. C 7 (27), 8344–8349. 10.1039/c9tc02457c

[B27] Tsai C.-T. Gottam S. R. Kao P.-C. Perng D.-C. Chu S.-Y. (2020). Improvement of OLED Performances by Applying Annealing and Surface Treatment on Electro-Deposited CuSCN Hole Injection Layer. Synth. Met. 269, 116537. 10.1016/j.synthmet.2020.116537

[B28] Wang H. Klubek K. P. Tang C. W. (2008). Current Efficiency in Organic Light-Emitting Diodes with a Hole-Injection Layer. Appl. Phys. Lett. 93 (9), 325. 10.1063/1.2978349

[B30] Wang M. Zhu W. Yin Z. Huang L. Li J. (2020). Synergistic Effects of Li-Doped NiO Film Prepared by Low-Temperature Combustion as Hole-Injection Layer for High Performance OLED Devices. Org. Electronics 85, 105823. 10.1016/j.orgel.2020.105823

[B29] Wang S. Qiao M. Ye Z. Dou D. Chen M. Peng Y. Wong W. Y. (2018). Efficient Deep-Blue Electrofluorescence with an External Quantum Efficiency beyond 10. Iscience 9, 532–541. 10.1016/j.isci.2018.10.026 30497025 PMC6258878

[B31] Wang S. Wu S. Ling Z. Chen H. Lian H. Portier X. (2020). Mechanically and Thermally Stable, Transparent Electrodes with Silver Nanowires Encapsulated by Atomic Layer Deposited Aluminium Oxide for Organic Optoelectronic Devices. Org. Electronics 78, 105593. 10.1016/j.orgel.2019.105593

[B32] Wen S.-W. Lee M.-T. Chen C. H. (2005). Recent Development of Blue Fluorescent OLED Materials and Devices. J. Display Technol. 1 (1), 90–99. 10.1109/jdt.2005.852802

[B33] Xie J. Wang X. Wang S. Ling Z. Lian H. Liu N. (2019). Solution-processed ZnO/MoS2 Quantum Dots Electron Extraction Layer for High Performance Inverted Organic Photovoltaics. Org. Electronics 75, 105381. 10.1016/j.orgel.2019.105381

[B34] Yadav R. A. K. Dubey D. K. Chen S. Z. Liang T. W. Jou J. H. (2020). Role of Molecular Orbital Energy Levels in Oled Performance. Scientific Rep. 10 (1), 1–15. 10.1038/s41598-020-66946-2 PMC7303122 32555238

[B35] Yang D. Y. Lee S.-M. Jang W. J. Choi K. C. (2014). Flexible Organic Light-Emitting Diodes with ZnS/Ag/ZnO/Ag/WO3 Multilayer Electrode as a Transparent Anode. Org. Electronics 15 (10), 2468–2475. 10.1016/j.orgel.2014.06.021

[B36] Yu S.-Y. Chang J.-H. Wang P.-S. Wu C.-I. Tao Y.-T. (2014). Effect of ITO Surface Modification on the OLED Device Lifetime. Langmuir 30 (25), 7369–7376. 10.1021/la4049659 24905669

[B37] Zhao B. Miao Y. Wang Z. Wang K. Wang H. Hao Y. (2016). High Efficiency and Low Roll-Off Green OLEDs with Simple Structure by Utilizing Thermally Activated Delayed Fluorescence Material as the Universal Host. Nanophotonics 6 (5), 1133–1140. 10.1515/nanoph-2016-0177

[B38] Zhao X. Chen J. Park N. G. (2019). Importance of Oxygen Partial Pressure in Annealing NiO Film for High Efficiency Inverted Perovskite Solar Cells. Sol. RRL 3 (4), 1800339. 10.1002/solr.201800339

[B39] Zhao Y. Wu S. Ling Z. Chen H. Yu N. Zhou P. (2020). Systematical Investigation of Ultrathin Doped Emissive Layer Structure: Achieving Highly Efficient and Long‐Lifetime Orange Organic Light‐Emitting Diodes. Adv. Mater. Inter. 7 (2), 1901609. 10.1002/admi.201901609

